# Temperature and Plant Genotype Alter Alkaloid Concentrations in Ryegrass Infected with an *Epichloë* Endophyte and This Affects an Insect Herbivore

**DOI:** 10.3389/fpls.2016.01097

**Published:** 2016-07-29

**Authors:** Louise M. Hennessy, Alison J. Popay, Sarah C. Finch, Michael J. Clearwater, Vanessa M. Cave

**Affiliations:** ^1^AgResearch Limited, Ruakura Research CentreHamilton, New Zealand; ^2^School of Science, University of WaikatoHamilton, New Zealand

**Keywords:** endophyte, *Epichloë festucae* var. *lolii*, AR37, epoxy-janthitrem, ryegrass, temperature, porina, bioactivity

## Abstract

Asexual *Epichloë* endophytes colonize agricultural forage grasses in a relationship which is mutually beneficial and provides the host plant with protection against herbivorous insects. The endophyte strain AR37 (*Epichloë festucae* var. *lolii*) produces epoxy-janthitrem alkaloids and is the only endophyte known to provide ryegrass with resistance against porina larvae (*Wiseana cervinata* (Walker)), a major pasture pest in cooler areas of New Zealand. This study examined the effect of temperature on concentrations of epoxy-janthitrems in AR37-infected ryegrass and determined how the resulting variations in concentration affected consumption, growth and survival of porina larvae. Twenty replicate pairs of perennial (*Lolium perenne* L.) and Italian ryegrass (*L. multiflorum* Lam.) plants with and without endophyte were prepared by cloning, with one of each pair grown at either high (20°C) or low (7°C) temperature. After 10 weeks, herbage on each plant was harvested, divided into leaf and pseudostem, then freeze dried and ground. Leaf and pseudostem material was then incorporated separately into semi-synthetic diets which were fed to porina larvae in a bioassay over 3 weeks. Epoxy-janthitrem concentrations within the plant materials and the semi-synthetic diets were analyzed by high performance liquid chromatography. AR37-infected ryegrass grown at high temperature contained high *in planta* concentrations of epoxy-janthitrem (30.6 μg/g in leaves and 83.9 μg/g in pseudostems) that had a strong anti-feedant effect on porina larvae when incorporated into their diets, reducing their survival by 25–42% on pseudostems. In comparison, *in planta* epoxy-janthitrem concentrations in AR37-infected ryegrass grown at low temperature were very low (0.67 μg/g in leaves and 7.4 μg/g in pseudostems) resulting in a small anti-feedant effect in perennial but not in Italian ryegrass. Although alkaloid concentrations were greatly reduced by low temperature this reduction did not occur until after 4 weeks of exposure. Alkaloid concentrations were slightly lower in Italian than in perennial ryegrass and concentrations were higher in the pseudostems when compared with the leaves. In conclusion, epoxy-janthitrems expressed by the AR37 endophyte show strong activity against porina larvae. However, when ryegrass plants are grown at a constant low temperature for an extended period of time *in planta* epoxy-janthitrem concentrations are greatly reduced and are less effective against this pasture pest.

## Introduction

Cool season grasses of the family Poaceae harbour fungal endophytes of the genus *Epichloë.* Asexual *Epichloë* endophytes grow as unbranched hyphae within the above ground tissues of the host plant and are transmitted between reproductive generations within the seed of its host. There is an ongoing debate over the nature of the relationship between endophytes and their host (Saikkonen et al., 1998, 2010). The relationship between agricultural forage grasses and asexual *Epichloë* endophytes, however, is thought to be defensive mutualistic. Defensive mutualism was first proposed by Clay (1988) and involves both organisms benefiting from the relationship. The endophyte gains from its host shelter, nutrients and a means of transmission (Saikkonen et al., 2004). In return the plant gains increased protection from biotic stresses including insects (Prestidge et al., 1982; Ball and Prestidge, 1992; Pennell et al., 2005; Popay et al., 2012), mammalian herbivores (Edwards et al., 1993; Cosgrove et al., 2002), pathogens (Pańka et al., 2013) and nematodes (Eerens et al., 1997; Bacetty et al., 2009) as well as increased tolerance to abiotic stresses such as drought and nutrient stress (Ravel et al., 1997; Kane, 2011; Nagabhyru et al., 2013).

Plants infected with an asexual *Epichloë* endophyte can have increased resistance against herbivorous insects due to the production of alkaloids which can have anti-feedant and/or toxic effects (Rowan et al., 1990; Jensen et al., 2009; Popay et al., 2009). Understanding bioactive alkaloids, their distribution within the plant and their effects on insects enables endophytes to be used in pest management strategies in both farming systems and turf. Fungal endophytes have been recognized as an important part of New Zealand’s pastoral sector since the early 1980s, as New Zealand contains a number of herbivorous pasture pests which can cause severe pasture damage.

The common toxic endophyte (*Epichloë festucae* var. *lolii*) strain found naturally infecting ryegrass (*Lolium perenne* and *L. boucheanum* syn. *L. hybridum*) in New Zealand produces alkaloids which provide the host with protection against a number of important pest insects (Prestidge et al., 1982; Popay and Baltus, 2001; Pennell et al., 2005). It also, however, produces lolitrem B an alkaloid which causes ryegrass staggers, a neurological impairment (Cunningham and Hartley, 1959; Fletcher and Harvey, 1981; di Menna et al., 2012) and the alkaloid ergovaline which causes vasoconstriction in grazing livestock (Dyer, 1993; Klotz et al., 2007). Due to these harmful effects on livestock endophyte research in New Zealand has focused on identifying different *E. festucae* var. *lolii* strains from European grasslands, where there is a greater chemical diversity, in an attempt to select those with a favorable chemical profile. Endophyte strains that are found to produce beneficial alkaloids, to deter insects, but not the detrimental alkaloids are then inoculated into New Zealand ryegrass cultivars (Johnson et al., 2013). These strains are known as ‘selected endophytes.’ One selected strain of *E. festucae* var. *lolii* is AR37. The only known alkaloids to be produced by AR37 are the epoxy-janthitrems (Tapper and Lane, 2004; Finch et al., 2007, 2012), a group of five compounds within the indole diterpene class of alkaloids. The epoxy-janthitrems are lipophilic compounds and are not easily translocated around the plant. Therefore, concentrations are thought to be highest in the pseudostem where endophyte mycelia are concentrated. AR37 provides ryegrass with protection against many of New Zealand’s major ryegrass pests including; African black beetle adults [*Heteronychus arator* (F.) Coleoptera: Scarabaeidae] (Ball et al., 1994), Argentine stem weevil larvae [*Listronotus bonariensis* (Kuschel), Coleoptera: Curculionidae] (Popay and Wyatt, 1995), root aphid [*Aploneura lentisci* (Passerini), Aphididae: Fordinae] (Popay et al., 2004; Popay and Gerard, 2007) and porina larvae (*Wiseana* spp. Hepialidae: Lepidoptera) (Jensen and Popay, 2004).

Porina are a group of seven closely related moth species endemic to New Zealand. The larvae of many of these species are a pest of cultivated grasses (Dugdale, 1994), particularly in the lower half of the North Island and in many parts of the South Island of New Zealand. Temperature is one of the main environmental factors which influences the location of porina in New Zealand. A study by Allan et al. (2002) looked at survival of larvae to pupation and then adulthood at four temperatures. Larval survival was found to be significantly lower when larvae were grown at 20°C compared to those grown at both 10 and 15°C. But survival was higher at 20°C than 5°C. Porina larvae are nocturnal and emerge at night from vertical burrows created beneath the soil surface (Barlow et al., 1986). Larvae can be highly destructive as they feed by cutting ryegrass tillers off at the base of the plant or by grabbing low lying leaves before dragging the herbage back into their burrow (Harris, 1969). The ‘selected’ endophyte AR37 has been shown to provide ryegrass with resistance against porina larvae in pot trials (Jensen and Popay, 2004), choice bioassays (Jensen and Popay, 2004) and field trials (Popay et al., 2012). In addition, when pure and semi-pure epoxy-janthitrem I, produced by AR37, was incorporated into a semi-synthetic diet and fed to porina larvae, larval diet consumption and growth were significantly reduced (Finch et al., 2010; Hennessy, 2015).

Several abiotic and biotic factors including plant growth temperature (Ball et al., 1995; Eerens et al., 1998; Salminen et al., 2005) and plant genotype (Adcock et al., 1997; Easton et al., 2002; Faeth et al., 2002) are known to affect alkaloid concentrations within endophyte-infected ryegrass. What effect these factors have on epoxy-janthitrem concentrations in ryegrass is not known. In this paper, the results of two experiments, a ryegrass pot trial and a porina larval bioassay, were designed to investigate the effect of high (20°C) and low (7°C) growth temperature on epoxy-janthitrem concentrations in AR37-infected perennial (*L. perenne* L.) and Italian (*L. multiflorum* Lam.) ryegrass and to examine how any resulting variations in concentration would affect consumption, growth and survival of porina larvae.

## Materials and Methods

### Establishment of Ryegrass Plants

Diploid perennial (cv ‘Grasslands Samson’) and Italian [cv ‘Grasslands Asset’ (PG255)] ryegrass plants were germinated from AR37-infected and endophyte-free seed in a Petri dish lined with moist filter paper. Germinated seedlings were sown into trays filled with potting mix (a commercial potting mix composed of N.Z. pine bark fines and fiber, pumice, coco fiber, controlled release fertilizer and a wetting agent (Daltons commercial)) on the 23rd of September (spring) and left to establish in a glasshouse. After seven and a half weeks plants were tested for endophyte infection using a tissue print immunoassay technique (Simpson et al., 2012). Thirty plants of each plant/endophyte treatment (AR37-infected perennial ryegrass, endophyte-free perennial ryegrass, AR37-infected Italian ryegrass and endophyte-free Italian ryegrass) were cloned (split in two) and planted into individual pots (12.5 cm by 10 cm) filled with potting mix (Daltons commercial). Plants were left to establish in a screenhouse for 16 weeks and were maintained with regular watering, trimming and fertilizing (1.8 g/L Thrive^®^ and 1.3 g/L urea).

### Establishment and Maintenance of a Porina Larval Colony

Forty female porina moths were collected in November–December 2013 from Allanton, near Mosgiel, in the South Island of New Zealand using an incandescent light as an attractant. Moths were held in 60 mL specimen vials overnight, to allow female moths to lay their eggs. The bursa copulatrix of the female moth was examined to determine the species of porina (Dugdale, 1994). Larvae from eggs laid by *Wiseana cervinata* moths were selected for this study. Porina eggs were sent to AgResearch, Ruakura Research Centre, Hamilton, New Zealand where they were surface sterilized with a copper oxychloride solution (Carpenter, 1983). Sterilized eggs were placed in a Petri dish lined with moist filter paper and left to hatch in an 18°C controlled environment (CE) room. Hatched larvae were placed into plastic rectangular containers (1000 mL) quarter filled with fine sized bark chips (40 larvae per container). Larvae were fed a semi-synthetic diet (Popay, 2001) which was cut into small pieces and evenly spread over the surface of the bark. Larvae were initially maintained at 15°C, but the temperature was later decreased to 7°C to slow larval growth. Larvae were maintained for 8 months with weekly diet changes.

### Effects of Temperature on Epoxy-Janthitrem Concentrations

The ryegrass pot trial contained eight treatments: Endophyte (AR37-infected or endophyte-free) × Temperature [high (20°C) or low (7°C)] × Plant species (Perennial or Italian ryegrass). Twenty healthy pairs of cloned plants from the original 30 cloned for each treatment were selected for the experiment. One of each cloned pair was randomly assigned to CE rooms, set at either 20 or 7°C with both set at a 12:12 h light:dark cycle. Plants were set up in identical randomized block designs in each room, with the same proximity to lights.

A herbage sample was taken from each plant at the beginning of the trial and after 4 weeks to compare changes in epoxy-janthitrem concentrations between treatments. At each of the two time points (Weeks 0 and 4) two tillers per plant were removed, the leaves and pseudostems (base of the plant to the first emerging leaf) were separated and material from five replicate plants combined to produce four replicate composite samples to be analyzed for epoxy-janthitrems. Immediately after samples were harvested they were put into sealed plastic bags and placed inside a chilly bin containing a cold pack. Samples were then frozen at -20°C approximately 20 min after harvest. After 10 weeks of growth in the CE rooms all plant material was harvested by replicate over a period of 2 weeks. Ryegrass was harvested by cutting all tillers off at the base of the plant; care was taken to ensure the meristem was included in the sample. Dead material was removed from the sample and live pseudostems and leaves were separated. All ryegrass samples taken were frozen soon after their harvest and later freeze dried and ground to a very fine powder. Total epoxy-janthitrem concentration (all five epoxy-janthitrem compounds) was determined by high performance liquid chromatography (HPLC).

To obtain a representative ryegrass sample of each treatment to be tested on porina larvae in the larval bioassay an approximately equal amount of ground plant material from the final harvest of each plant in a treatment (20 plants) was combined and mixed thoroughly. Three samples (3 g each, one for each week of the 3 weeks porina larval bioassay) of plant material from each treatment were weighed into separate glass vials and set aside for use in the porina larval bioassay.

### Larval Bioassay

Plant material harvested from the eight treatments in the pot trial described above was fed to porina larvae in a bioassay. Tillers were separated into pseudostems and leaves and were tested separately to give a total of 16 treatments, with 12 replicate larvae per treatment. Porina larvae (32 weeks old), weighing between 226 and 692 mg, were selected from 27 parent moths. Larvae were removed from their containers and starved overnight before being weighed and assigned to a replicate so that larvae within a replicate were of similar weight. Within each replicate, larvae were randomly allocated to a treatment. Individual larvae were then placed into specimen containers (150 mL polystyrene) three quarters full with fine sized bark chips. Larvae were fed plugs (14–15 mm diameter cut with a cork borer, average weight of 788 mg) of a semi-synthetic diet containing ground plant material from each of the treatments. Fresh diets were made weekly and diets changed over in each larval container on days 4 and 7 of each week. Diets were kept at 4°C between diet changes. Consumption was estimated by change in diet weight between diet changes. Larvae were checked for survival at each diet change and weighed again after 3 weeks at the completion of the trial. Total epoxy-janthitrem concentration in fresh diets and remnant diets (diets larvae had fed on for 3–4 days) were determined by HPLC.

The insect bioassay was conducted in a CE room at 15°C. Specimen containers were placed into polystyrene trays that were covered with black polythene to exclude light. These conditions were necessary as epoxy-janthitrems degrade when exposed to light.

### Semi-Synthetic Diet

Fresh carrot (500 g) was blended with Milli-Q water (1000 mL) and strained to obtain carrot juice (750 mL). Carrot juice was mixed with agar (18 g) and warmed in a microwave until boiling point. Diet was kept warm in a water bath, to prevent agar setting, while individual diets were made. Sixteen batches of diet (27 mg) were weighed out separately into warm glass beakers. One of the ground ryegrass samples (3 g) was added to each beaker, mixed thoroughly and then poured into a Petri dish and smoothed flat. Petri dishes were wrapped in tin foil to exclude light.

### Alkaloid Analyses

Epoxy-janthitrem concentrations in both herbage and diet samples were quantified by HPLC. Epoxy-janthitrems were extracted from ground herbage (20 mg) or diet samples (50 mg) with water-acetone (1:4, 1 mL) using an over-over mixer at 30 rotations/min for 1 h. The extract was then centrifuged (1 min, 5600 *g*, Eppendorf, Hamburg, Germany) and analyzed by HPLC. Epoxy-janthitrems were quantified by comparison with a reference standard (N-benzyl-1, 8-naphthaleneimide, 5 μg/mL) which had previously been compared with a pure epoxy-janthitrem I standard (Finch et al., 2012, 2013). Due to the instability of epoxy-janthitrems the use of an epoxy-janthitrem standard is not practical for routine analysis. Samples were protected from light during extraction and analysis. For analysis of extracts a 4.6 mm × 250 mm ODS C18 column (Phenomenex, Torrance, CA, USA) fitted with a 4 mm × 3 mm Phenomenex Security GuardTM containing two C18 cartridges (Torrance, CA, USA) was used with an eluent of water-acetonitrile (1:19, 1 mL/min). Eluting compounds were detected with an Agilent Series 1100 fluorescence detector (excitation at 333 nm, emission detection at 385 nm).

### Statistical Analyses

Data on epoxy-janthitrem concentration, larval diet consumption, mortality and growth collected during the bioassay were analyzed using GenStat 16th and/or 17th edition. Epoxy-janthitrem concentrations in ryegrass plants at the beginning of the trial, after 4 weeks and after 10 weeks of growth in the CE rooms were analyzed using 3-way analysis of variance (ANOVA) blocked by replicate, with treatment factors Temperature, Plant species, and Plant part. All variables were natural log transformed prior to analysis to stabilize the variance. Larval diet consumption data (average diet consumed per day) were analyzed using a REML linear mixed model, with replicate a random effect, with fixed effects of Endophyte by Temperature by Species by Plant part. To take into account the higher variance of data from the AR37 high temperature treatments compared with data from low temperature treatments, a separate residual variance was defined for the AR37 high temperature treatments. Larval growth data (not transformed) were analyzed using 4-way ANOVA blocked by replicate, with treatment factors Endophyte, Temperature, Species, and Plant part. In all analyses differences were compared using protected Fisher’s least significant difference *post hoc* tests, conducted at the 5% significance level.

## Results

### Effects of Temperature on Epoxy-Janthitrem Concentrations

**FIGURE 1:**
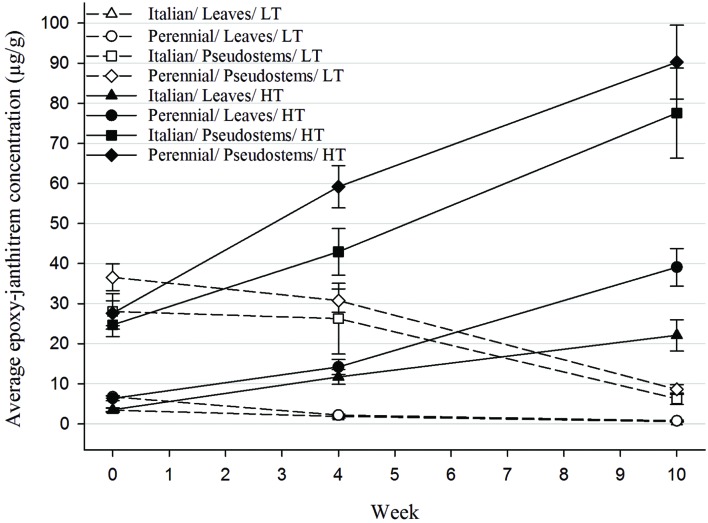
***In planta* epoxy-janthitrem concentrations (μg/g) for each of the AR37-infected ryegrass treatments at week 0 (sample 1), week 4 (sample 2), and week 10 (final harvest) (error bars are ±SEM of raw data).** HT, high temperature (20°C); LT, low temperature (7°C).

Epoxy-janthitrem concentrations within the leaves and pseudostems of AR37-infected Italian and perennial ryegrass were determined at the beginning of the trial and then after 4 and 10 weeks to monitor changes in concentration over time at the different temperatures (**Figure 1**). When ryegrass was grown at high temperature (HT) epoxy-janthitrem concentrations were greatly increased. Concentrations were 2–3 times higher than the initial concentrations after 4 weeks and 3–7 times higher after 10 weeks. In contrast to this, concentrations declined in ryegrass pseudostems grown at low temperature (LT) although the decrease was small over the first 4 weeks.

After 10 weeks epoxy-janthitrem concentrations were highly variable among treatments and plants within a treatment especially in the two high temperature pseudostem treatments, which contained high epoxy-janthitrem concentrations.

On average, epoxy-janthitrem concentrations at the beginning of the trial were significantly higher (*P* < 0.05) in perennial ryegrass than in Italian ryegrass and this difference was maintained throughout the trial (**Table 1**). Concentrations were also significantly higher (*P* < 0.05) in the pseudostems when compared with the leaves of ryegrass plants at all three sample points. An interaction between Species and Plant part was significant at the beginning of the trial. In this interaction epoxy-janthitrem concentrations in perennial ryegrass leaves were significantly higher than those in Italian leaves. But there was no significant difference between perennial and Italian pseudostems. Temperature and the Temperature by Plant part interaction had a highly significant (*P* < 0.001) effect on epoxy-janthitrem concentration after 4 and 10 weeks, with concentrations significantly higher in pseudostems grown at high temperature.

**Table 1:** *P*-values for the effects of Temperature (high and low), Species (perennial and Italian), Plant part (pseudostems and leaves) and their interactions from the analysis of epoxy-janthitrem concentration in ryegrass at the beginning of the trial, after 4 weeks and after 10 weeks of growth in the controlled environment rooms.

	*P*-value		
Source of variation	Week 0 (Sample 1)	Week 4 (Sample 2)	Week 10 (Final harvest)
Species	**<0.001**	**0.029**	**<0.001**
Plant part	**<0.001**	**<0.001**	**<0.001**
Temperature	0.181	**<0.001**	**<0.001**
Species × Plant part	**0.005**	0.523	0.429
Temperature × Plant part	0.205	**<0.001**	**<0.001**
Species × Temperature	0.315	0.884	0.701
Species × Temperature × Plant part	0.849	0.877	0.089

*Bold values are the statistically significant (P < 0.05) values.*

### Larval Bioassay

There were statistically significant effects of Endophyte, Temperature, Plant species, and Plant part on both larval diet consumption and larval growth (**Table 2**).

**Table 2:** Interactions between Endophyte (AR37 or endophyte-free), Temperature (high and low), Species (perennial and Italian), and Plant part (pseudostems and leaves) for larval diet consumption and larval growth data within the larval bioassay.

	*P*-value	
Source of variation	Diet consumption	Larval growth
Endophyte	**<0.001**	**<0.001**
Temperature	**<0.001**	**<0.001**
Plant part	**<0.001**	**<0.001**
Species	0.866	0.994
[1.693pt] Endophyte × Species	**0.005**	**0.006**
Endophyte × Temperature	**<0.001**	**<0.001**
[1.693pt] Species × Temperature	0.996	0.224
Endophyte × Plant part	0.056	**0.002**
Species × Plant part	0.461	0.597
Temperature × Plant part	**0.006**	**<0.001**
Endophyte × Species × Temperature	**0.033**	**0.002**
Endophyte × Species × Plant part	**0.006**	**0.022**
Endophyte × Temperature × Plant part	0.316	**0.022**
Species × Temperature × Plant part	0.989	0.656
Endophyte × Species × Temperature × Plant part	0.170	0.112

*Bold values are the statistically significant (P < 0.05) values.*

**FIGURE 2:**
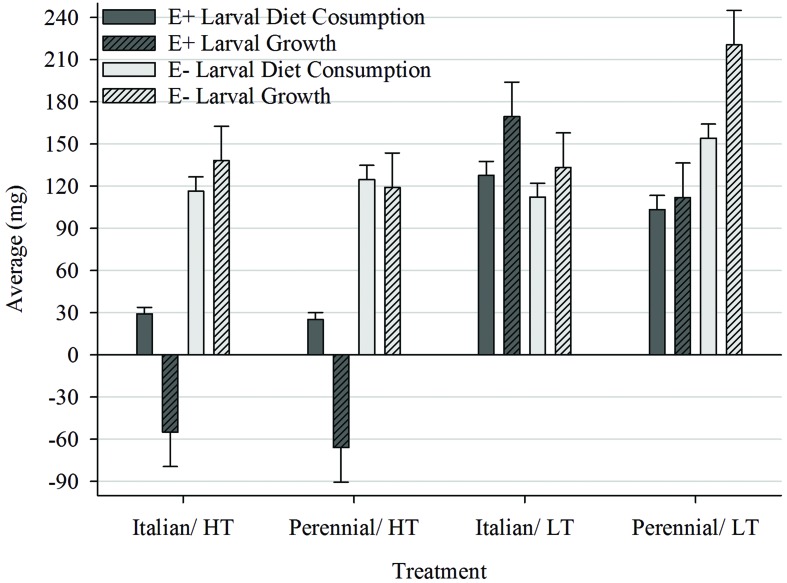
**Comparison of average diet consumption (mg; ±SE) and average larval growth (mg; ±SED) within the Endophyte (E+ = AR37-infected or E- = endophyte-free) × Temperature [HT = high (20°C) or LT = low (7°C)] × Species (perennial or Italian) interaction**.

Larvae fed AR37-infected (E+) ryegrass grown at HT consumed significantly (*P* < 0.05) less diet and gained significantly less weight than larvae fed E+ ryegrass grown at LT and endophyte-free (E-) ryegrass at both temperatures (**Figure 2**). In the LT treatment, however, only larvae fed E+ perennial ryegrass consumed less diet (*P* < 0.05) and gained less weight (*P* < 0.05) than larvae in the equivalent E- treatment with no differences for the Italian ryegrass. In E- perennial ryegrass treatments significantly more diet was consumed and larval growth was higher in the LT treatment than the HT treatment. No such difference was found in the corresponding Italian ryegrass treatments. When comparing perennial with Italian treatments grown at LT larvae fed E- ryegrass consumed more and gained more weight on perennial. In contrast, when fed E+ ryegrass there was no difference (*P* < 0.05) in consumption but larvae gained significantly more weight on Italian.

**FIGURE 3:**
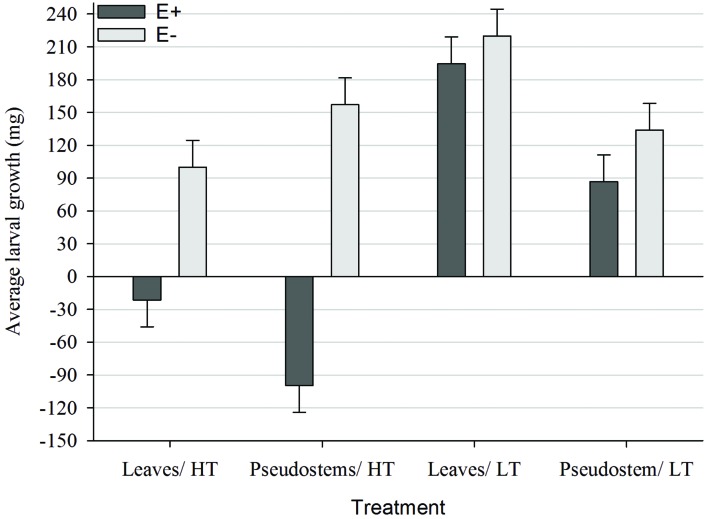
**Comparison of average growth (mg; ±SED) within the Endophyte (E+ = AR37-infected or E- = endophyte-free) × Temperature [HT = high (20°C) or LT = low (7°C)] × Plant part (pseudostems or leaves) interaction**.

Both pseudostems and leaf blades from E+ plants grown at HT caused larvae to lose weight, with pseudostems having a significantly greater (*P* < 0.05) effect than leaf blades (**Figure 3**). In comparison, all larvae fed E+ ryegrass grown at LT gained weight but those fed pseudostems gained less weight (*P* < 0.05) than those fed leaf blades. There was no significant difference (*P* > 0.05) in growth between larvae fed E+ ryegrass grown at LT and the equivalent E- treatment, for both pseudostems and leaves. Larvae gained more weight (*P* < 0.05) when fed E- ryegrass pseudostems than leaves from plants grown at HT whereas the opposite occurred for the LT E- plants.

The greatest larval mortality occurred in the HT pseudostem treatments where larval mortality was 41.7% in the perennial ryegrass treatment and 25% in the Italian. Mortality in all remaining treatments was less than 8.3%.

### Epoxy-Janthitrem Concentrations within Insect Diets

Epoxy-janthitrem concentrations were analyzed by HPLC in freshly prepared diet (day 0), diet added to containers on day 4 (stored at 4°C from days 0 to 4) and in remnant diets (recovered from insect containers on days 4 and 7) to ensure the fresh diet concentrations were similar at each diet change and to check that the concentrations in the diet were not substantially degraded when diet plugs were exposed to porina larvae. Epoxy-janthitrem concentrations in diet added to containers on day 4 were not substantially different (average 10.7%) from fresh diet concentrations (**Table 3**). Furthermore, epoxy-janthitrem concentrations were not substantially degraded (average 9.1%) during the time diets were in the insect trial. At the end of the trial, samples of the endophyte-free diets (week 3) were analyzed for epoxy-janthitrem to confirm that there was no contamination. No epoxy-janthitrems were found.

**Table 3:** Average epoxy-janthitrem (EJ) concentration (μg/g) in fresh diets, the range and estimated dry weight concentrations of epoxy-janthitrem (μg/g).

Ryegrass species	Temperature	Plant part	Average EJ concentration (μg/g)	Range	Estimated dry weight conc. (μg/g)
Italian	Low	Leaves	0.08	0.07–0.10	0.66
Italian	Low	Pseudostems	0.85	0.82–0.88	7.02
Perennial	Low	Leaves	0.10	0.09–0.10	0.83
Perennial	Low	Pseudostems	1.62	1.59–1.65	13.38
Italian	High	Leaves	2.33	2.27–2.40	19.24
Italian	High	Pseudostems	11.14	11.02–11.31	91.99
Perennial	High	Leaves	3.78	3.60–3.93	31.21
Perennial	High	Pseudostems	13.68	12.89–14.18	112.96

*Wet weight-dry weight conversion = 8.258.*

## Discussion

This experiment has shown that when AR37-infected ryegrass was grown at 20°C epoxy-janthitrem concentrations were greatly increased, resulting in a strong anti-feedant effect on porina larvae that led to high weight loss and in the case of pseudostems, increased mortality. In contrast, epoxy-janthitrem concentrations declined markedly in plants grown at 7°C causing low level deterrence and a small reduction in weight gain of larvae fed perennial ryegrass. Although epoxy-janthitrem concentrations were greatly reduced by low temperature this reduction did not occur until after 4 weeks of exposure.

When fed to larvae E+ perennial ryegrass grown at LT reduced larval consumption and growth but Italian ryegrass did not. This is likely explained by the higher epoxy-janthitrem concentrations in perennial ryegrass insect diets, although this effect was exaggerated by the large increase in consumption and growth of larvae fed E- perennial ryegrass (cv ‘Grasslands Samson’) that did not occur in larvae fed E- Italian ryegrass (cv ‘Grasslands Asset’). It is possible that differences in the ratios of the five epoxy-janthitrem compounds between perennial and Italian ryegrass may have contributed to the differences in bioactivity, particularly if certain compounds, or combinations of compounds are more bioactive than others. It is also possible that there was an unknown alkaloid produced in higher concentrations in perennial than Italian ryegrass.

Results from this study have shown an anti-feedant effect of the endophyte AR37 on porina larvae when ground herbage was incorporated into an insect diet. Epoxy-janthitrems within AR37 are likely to be responsible for this bioactivity as pure and semi-pure epoxy-janthitrem I have previously been shown to have an anti-feedant effect on porina when incorporated into semi-synthetic diets (Finch et al., 2010; Hennessy, 2015).

Although the results from this experiment clearly show an anti-feedant effect of AR37 it could not be determined whether this endophyte also has a toxic effect on larvae. Here toxicity is defined as a reduction in growth and survival of an insect above that which can be attributed to starvation. Pseudostems of AR37-infected ryegrass grown at HT, which contained the highest epoxy-janthitrem concentrations, reduced larval survival. A reduction in survival could indicate toxicity but it is also possible that larvae may have died due to starvation caused by the strong anti-feedant effect of AR37. Further research is required to resolve this.

Plant growth temperature is known to affect the concentrations of other important endophyte alkaloids. Seasonal concentrations of lolitrem B, which like the epoxy-janthitrems is in the indole diterpene class of alkaloids, and peramine were monitored by Ball et al. (1991). Lolitrem B concentrations were found to be highest during the summer months and lowest during the winter when rainfall is higher and temperatures are cooler. Peramine concentrations were comparatively stable, but were also significantly lower during winter when compared to summer and autumn. Although caution must be applied when relating results of pot trials to field conditions the results of this study suggest that epoxy-janthitrems could respond to temperature in a similar way. However, for epoxy-janthitrem concentrations to decrease to the low levels observed in this experiment plants would have to be exposed to constant low temperatures for an extended period of time (at least 4 weeks). Under field conditions temperatures will constantly fluctuate which may mean that epoxy-janthitrem concentrations are not decreased to the extent as that observed in this study.

The reduction in epoxy-janthitrem concentrations in plant material grown at low temperatures suggests that AR37 may not provide the highest level of protection against porina larvae during the winter months in parts of New Zealand. Porina are major pasture pests particularly in the southern areas of both the North and South Island of New Zealand where they are capable of causing severe pasture damage. Several species of porina are known pasture pests, the moths of which have different peak flight periods. Moths of *W. cervinata*, the species tested in this experiment, fly between October and December in the South Island (Barratt et al., 1990). Young larvae of this species will begin feeding on ryegrass during the late spring and summer months, when temperatures are warm. Results from this study suggest that during this period AR37-infected ryegrass is likely to contain relatively high epoxy-janthitrem concentrations which should provide good control over larvae. Larvae of the later flying species, *W. copularis*, which can fly as late as February (Barratt et al., 1990) begin feeding on AR37-infected ryegrass when temperature and alkaloid concentrations are likely to be lower and less effective at controlling larval populations.

The mechanisms by which temperature and plant genotype affected alkaloid concentrations in perennial and Italian ryegrass plants in this study are not known. These factors may have indirectly affected alkaloid concentrations by influencing the ratio of endophyte mycelial biomass to plant biomass, resulting in changes in alkaloid concentration (di Menna and Waller, 1986; Breen, 1992; di Menna et al., 1992; Ju et al., 2006). Alternatively, alkaloid biosynthesis, metabolism, or degradation rates may have been directly affected by temperature or plant genotype (Spiering et al., 2005).

No published information is available comparing epoxy-janthitrem concentrations in the leaves and pseudostems of AR37-infected ryegrass plants. In this study, concentrations were found to be markedly higher in the pseudostems than the leaves at both temperatures and for both cultivars. This distribution is not uncommon and has also been found for lolitrem B (di Menna et al., 1992; Davies et al., 1993; Keogh et al., 1996; Ball et al., 1997). Alkaloids such as lolitrem B and the epoxy-janthitrems are lipophilic compounds and are not easily translocated around the plant (Ball et al., 1993; Munday-Finch and Garthwaite, 1999; Spiering et al., 2005) thus distribution tends to be similar to that of the endophyte, which is generally higher in the pseudostem and lower in the leaves (Musgrave, 1984; Musgrave and Fletcher, 1984; Keogh and Tapper, 1993). Maintaining high alkaloid concentrations in the pseudostem is advantageous for both the host plant and the endophyte as the meristem, the tissue containing undifferentiated cells and where growth occurs is located at the base of the ryegrass plant (Popay, 2009). Tiller death will occur if an insect severely damages the meristem. Insect damage to the leaves of ryegrass plants is not as harmful to the plant itself, as ryegrass is adapted to animal grazing (Popay, 2009). However, the more leaf material the insect is able to consume the less that is available for both plant photosynthesis and consumption by grazing livestock, resulting in reduced plant growth and animal productivity.

The endophyte AR37 is very important for the control of porina in New Zealand as although other endophytes such as AR1 and the common toxic strain provide protection against some pest insects (Prestidge et al., 1982; Popay et al., 1999; Pennell et al., 2005; Popay and Gerard, 2007; Popay and Thom, 2009) it is only AR37 which provides ryegrass with protection against porina (Jensen and Popay, 2004; Popay et al., 2012). Control against porina, which are a major pasture pest in parts of New Zealand, currently involves an integrated pest management strategy involving planting ryegrass infected with the AR37 endophyte and the application of insecticides at particular times of the year (Barratt et al., 1990). The results of this paper support the continued use of integrated pest management strategies to control porina populations in the field.

Leading on from this study field trials should be conducted to determine how temperature affects epoxy-janthitrem concentrations in AR37-infected ryegrass in the field and how these concentrations then impact on porina populations. If concentrations are found to be reduced under certain environmental conditions the next step could be to identify existing ryegrass cultivars and/or plant genotypes, from which a new breeding line could be produced, that produces higher alkaloid concentrations when grown at low temperature.

## Author Contributions

LH carried out this research as a part of her Masters of Science (Research). AP was a co-supervisor and the main supervisor of all experimental work. SF was a co-supervisor and oversaw all of the chemical analyses. MC was the University supervisor and VC provided statistical expertise.

## Conflict of Interest Statement

AP is a patent holder for AR37; AP and SF receive research funding from IP owners Grasslanz Technology Ltd. and licensee, PGG Wrightson Seeds. AP receives a share of royalties from the sale of AR endophytes. All the other authors declare that the research was conducted in the absence of any commercial or financial relationships that could be construed as a potential conflict of interest.

## Abbreviations

HT: high temperature (20°C)
LT: low temperature (7°C)
